# The role of pyroptosis in the occurrence and development of pregnancy-related diseases

**DOI:** 10.3389/fimmu.2024.1400977

**Published:** 2024-09-16

**Authors:** Jiahui Li, Min Wang, Haiyan Zhou, Zhong Jin, Haonan Yin, Shuli Yang

**Affiliations:** Department of Gynecology and Obstetrics, The Second Hospital of Jilin University, Changchun, Jilin, China

**Keywords:** cell death, pyroptosis, pregnancy complications, preeclampsia, GDM

## Abstract

Pyroptosis is a form of programmed cell death that is crucial in the development of various diseases, including autoimmune diseases, atherosclerotic diseases, cancer, and pregnancy complications. In recent years, it has gained significant attention in national and international research due to its association with inflammatory immune overactivation and its involvement in pregnancy complications such as miscarriage and preeclampsia (PE). The mechanisms discussed include the canonical pyroptosis pathway of gasdermin activation and pore formation (caspase-1-dependent pyroptosis) and the non-canonical pyroptosis pathway (cysteoaspartic enzymes other than caspase-1). These pathways work on various cellular and factorial levels to influence normal pregnancy. This review aims to summarize and analyze the pyroptosis pathways associated with abnormal pregnancies and pregnancy complications. The objective is to enhance pregnancy outcomes by identifying various targets to prevent the onset of pyroptosis.

## 1 Introduction

**Figure 1:**
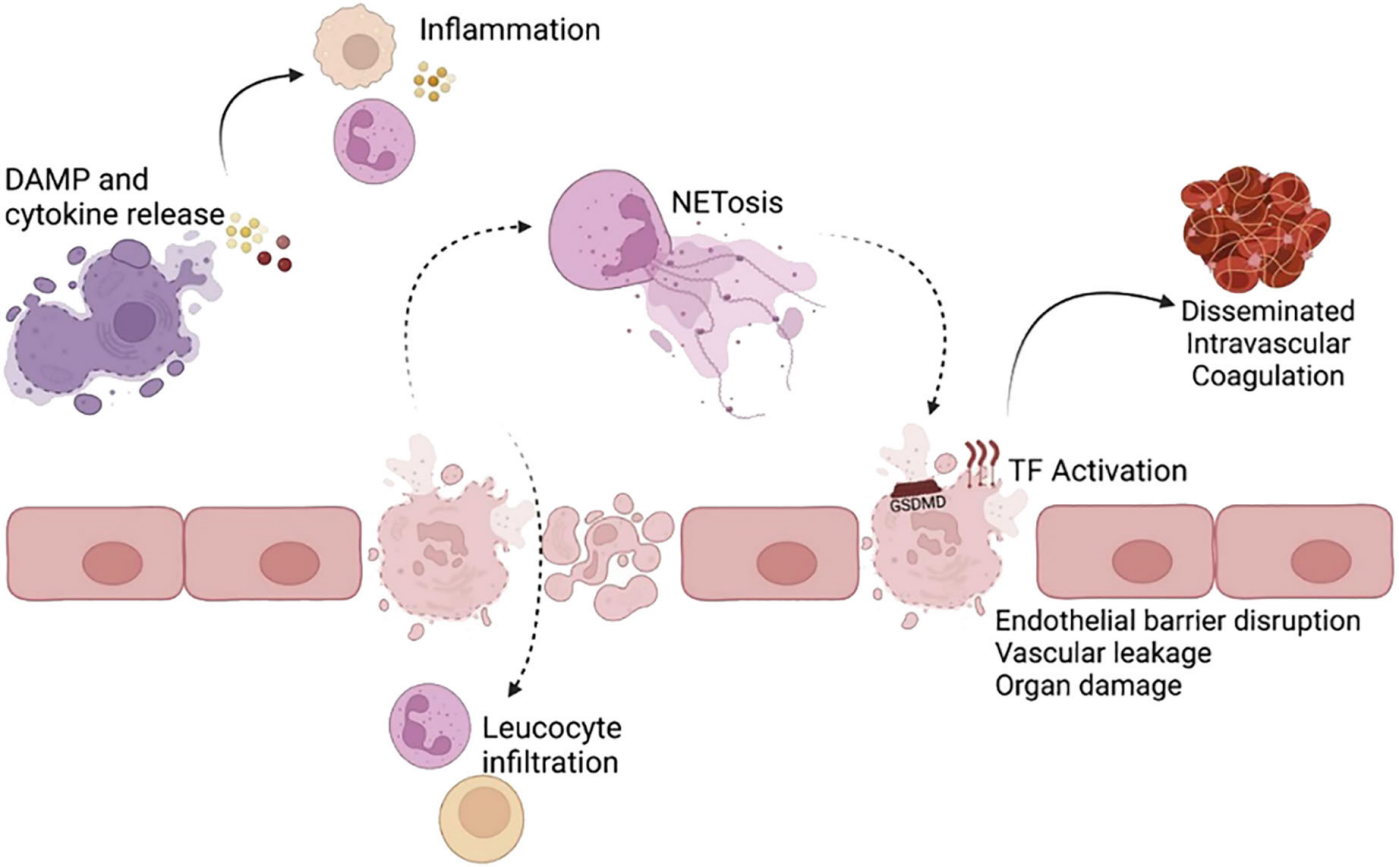
Inflammatory and pathological consequences of pyroptosis (1). Pyroptosis, induced by Gasdermin D (GSDMD) pore formation, results in the release of proinflammatory cytokines, alarmins, and damage-associated molecular patterns (DAMPs). These inflammatory molecules act on bystander cells (e.g., endothelial cells, lymphocytes) to promote an inflammatory response. The resulting cell death and inflammation can disrupt the endothelial barrier in blood vessels and vital organs, such as the lungs, leading to leucocyte infiltration. GSDMD pore formation activates the coagulation cascade and can contribute to lethality from disseminated intravascular coagulation. Pyroptosis in neutrophils and other cells triggers NETosis. Improper neutrophil extracellular trap (NET) and aberrant NETosis and DAMP removal can further induce pyroptosis and tissue damage. Reproduced with permission from [Vasudevan SO, Behl B, Rathinam VA], [Pyroptosis-induced inflammation and tissue damage.]; published by [Semin Immunol], [2023].

Cell death is typically classified as either non-programmed or programmed. Pyroptosis is a lytic form of programmed cell death (1). Pyroptotic cells undergo membrane blistering, swelling, and flattening before lysis, as opposed to the explosive rupture observed in necroptotic apoptotic cells or the shrinking and wilting of apoptotic cells (2). This process results in the formation of cellular pores, membrane ruptures, cellular swelling, and the release of cellular contents. It also leads to DNA fragmentation, cellular vesiculation, and the formation of pyknotic vesicles (3, 4). Pyroptosis can expose microorganisms, eliminate pathogens, or retain pathogens within the burnt carcass to promote a local inflammatory response (5, 6). Pyroptosis can have both beneficial and detrimental effects on the host, depending on the degree and context of activation (1). In normal physiology, pyroptosis plays a crucial role in defending the host against pathogen infections. However, excessive pyroptosis can lead to maladaptive and prolonged inflammatory responses that are implicated in the development of diseases (3). Emerging evidence suggests that pyroptosis contributes to the pathogenesis of a wide range of non-infectious diseases, including aseptic inflammatory diseases, autoimmune disorders, neurological disorders, cancer, atherosclerosis, acute injury, and adverse pregnancy complications (7–10) (see **Figure 1**).

## 2 The model of pyroptosis

**Figure 2:**
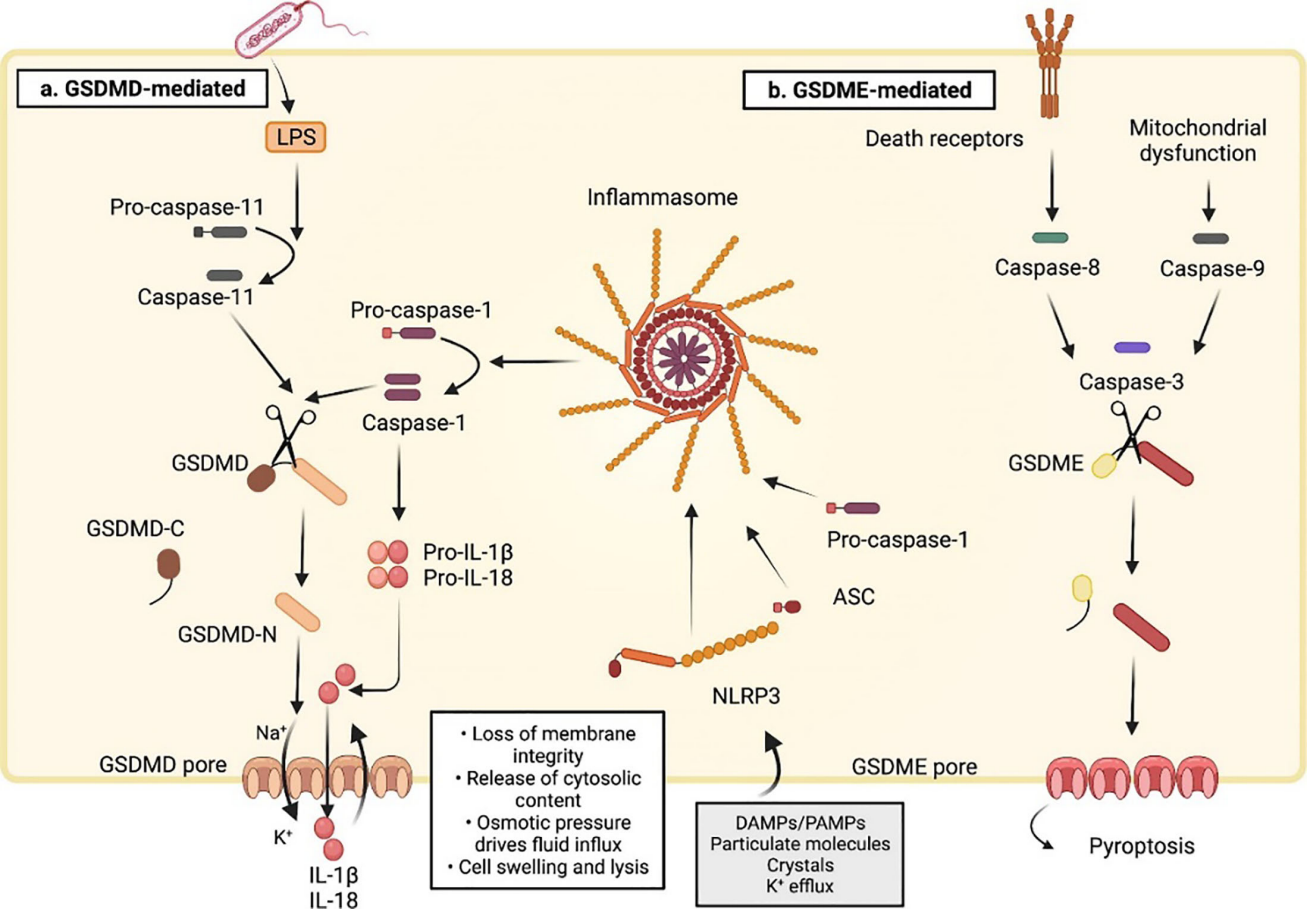
Molecular mechanisms of pyroptotic cell death (16). **(A)** The sensing of cytosolic disturbances by NLRP3 (NLR family pyrin domain-containing 3) receptor recruits the adaptor protein apoptosis-associated speck-like protein containing (ASC) to a large aggregate platform (called the inflammasome) that serves as a site of caspase-1 activation. Active caspase-1 cleaves the 53-kDa GSDMD and generates a 31-kDa N-terminal pore-forming fragment that controls pyroptosis(Canonical model). GSDMD can also be processed via inflammasome-independent activation of caspase-11(Non-canonical model). In this pathway, caspase-11 in mice and caspase-4 and 5 in humans bind to and are activated by LPS released into the cytoplasm after gram-negative bacterial infection. Unlike cleaved caspase-1, cleaved caspase-11 does not convert pro-IL-1β and pro-IL-18 to their mature forms. Instead, the process of pyroptosis promotes K+ efflux to activate caspase-1-dependent maturation of the pro-inflammatory cytokines IL-1β and IL-18 after NLRP3 inflammasome activation, leading to cellular pyroptosis. **(B)** Besides GSDMD, there is also a gasdermin E (GSDME)-dependent pyroptosis. GSDME is cleaved by caspase-3 upon mitochondrial dysfunction or death receptor activation. The GSDME N-fragments promote cell swelling and lysis by forming pores in the plasma membrane. ASC, apoptosis-associated speck-like protein containing; LPS: lipopolysaccharide. Reproduced with permission from [Ketelut-Carneiro N, Fitzgerald KA. Apoptosis], [Pyroptosis, and Necroptosis-Oh My! The Many Ways a Cell Can Die.]; published by [J Mol Biol], [2022].

In 2015, the discovery and characterization of GSDMD proteins (14) led to the identification of a fundamental mechanism underlying cellular pyroptosis (11). GSDMD is encoded by a gene on chromosome 8q24.3 and is the primary execution gene for inflammatory vesicle-driven pyroptosis (12). It is widely expressed in various tissues (colon, liver, brain and so on) and immune cells (13–15). Pyroptosis is carried out by pore-forming proteins called gasdermins (1), which are triggered by pyroptotic caspases (caspase-1/4/5/11) (4, 11). The initiating event of pyroptosis is the activation of the inflammasome, a multi-protein complex that activates caspase-1 (16). Due to the involvement of various cysteoaspartic enzymes, pyroptosis has recently been defined as “gasdermin-mediated programmed necrotic cell death”, as gasdermin activation and pore formation in cell membranes are common features of both canonical (caspase-1-dependent pyroptosis) and non-canonical (cysteoaspartic enzymes other than caspase-1) pyroptosis (17). In the non-canonical pathway, caspase-4/5 (in human) and caspase-11 (in mouse) are directly activated by their lipopolysaccharide (LPS) ligands (11), and upon oligomerization of the LPS-cysteaspase complex, cysteaspase-4/5/11 act as effector proteins, cleaving full-length GSDMD and triggering pyroptosis (18). Other proteins have also been proposed to cleave GSDMD, including cysteinyl asparagine-8, neutrophil elastase (NE) and histone G (19–23). Activated caspase-4/5/11 cleaves the junctional region of GSDMD to release its N-terminal pore-forming structural domain (GSDMD-NT), which oligomerizes in the plasma membrane to form a pore that allows the release of DAMPs, such as the mature form of interleukin-1β (IL-1β), which recruits immune cells and induces inflammation (24), alters the intracellular environment and ultimately leads to cell death (11, 25–27) (see **Figure 2**). Since then, the GSDMD has been considered to be a central factor in the execution of cellular cell death (11).

GSDMs are expressed in a wide range of cell types and tissues (17, 28–30), and GSDMD is only one member of the GSDM family, which is evolutionarily and structurally conserved (16). Human GSDMs include GSDMA (31, 32), GSDMB (32), GSDMC (32, 33), GSDMD (34), GSDME and pejvakin(PJVK), also known as DFNB59 or GSDMF (35), a GSDM-related gene common to both humans and mice (16, 36). Mice lack GSDMB but have three homologs encoding GSDMA (GSDMA 1-3), four homologs of GSMDC (GSDMC 1-4) and GSDMD, GSDME (37) and PJVK. Except PJVK, the gasdermin family of proteins consists of two conserved structural domains, the N-terminal pore-forming structural domain, and the C-terminal deterrent protein structural domain (38, 39), which are linked by a variable linkage region (36). The C-terminal structural domain has an autoinhibitory function and is removed by proteolytic cleavage of the linker region, mediated by enzymes such as cysteinyl asparaginase (36). PJVK is a structurally unique exception to this rule, lacking a cleavable linker and containing only a truncated C-terminal structural domain (40, 41).

Gasdermin D cleavage has both positive and negative regulators of pore formation (42). These pores act as conduits for inward and outward flow across the cell membrane. Living cells employ active transport to uphold an ion gradient across the plasma membrane. This gradient collapses upon pore formation (42). Gasdermins exhibit structural similarity, and it has been hypothesized that pores formed by other members of the Gasdermin family may result in comparable intracellular consequences. However, there is currently insufficient data to directly support this hypothesis (42).

Pathogen-associated molecular patterns (PAMPs), present on the surface of toxins, viruses, and bacteria, or DAMPs produced following tissue or cellular injury, can be recognized by intracellular pattern recognition receptors (PRRs) (43, 44). Nucleotide-binding oligomerization domain-like receptors (NLRs), leucine-rich repeat (LRR) receptors, and NOD-like receptor pyrin-containing 3 (NLRP3) inflammasomes are key mediators of sterile inflammation induced by various types of DAMPs (45). Unlike other modes of cell death, pyroptosis is characterized by caspase-1-dependent plasma membrane rupture, as well as the facilitation of NLRP3 inflammatory vesicles, and the release of DAMPs and cytokines. It is important to note that all evaluations presented are objective and supported by evidence. The cytokines such as IL-1β and interleukin-18(IL-18) are released into the extracellular environment, leading to sterile inflammation (46–49). This can induce a range of diseases. Pyroptosis mediated by the NLRP3 inflammasome can also be stimulated by pathogen-associated molecules such as LPS, flagellin, or DNA fragments, as well as by hazard-associated molecules such as reactive oxygen species (ROS) (50). These are elements of the atypical pathway.

Disruption of immune function can lead to infertility, placental inflammation, and pregnancy complications such as PE, maternal obesity, gestational diabetes mellitus (GDM), spontaneous abortion, and recurrent miscarriage (RM) (51). Excessive inflammation has been shown to be a potential cause of pregnancy complications such as PE and miscarriage (52). In this paper, we review the mechanisms and pathophysiology of pyroptosis leading to various pregnancy complications.

Furthermore, cellular death occurs via sensor proteins that detect pathogens and serve as a platform for the recruitment and activation of Caspase-1. Caspase-1 then cleaves the cytokines IL-1β and -18, as well as the pore-forming protein GSDMD. In comparison, apoptosis inhibitory protein (NAIP) and NLR family, CARD domain-containing protein 4 (NLRC4) function as a sensor (NAIP) and junctional protein (NLRC4), respectively, to form individual inflammasomes in a synergistic manner. It seems that NAIP/NLRC4 inflammasomes serve to safeguard mucosal barriers, including the lungs, stomach, and intestines, from bacterial pathogen invasion. Upon systemic activation, NAIP/NLRC4 results in a robust autoinflammatory response that is deleterious to the host (53). For example, in response to Salmonella, human intestinal epithelial cells undergo caspase-4-dependent pyroptosis, IL-18 cytokine release, restriction of bacterial replication, and extrusion of infected cells (54). The intrinsic expression of NAIP or NLRC4 in intestinal epithelial cells is both necessary and sufficient for limiting the intraepithelial Salmonella load, indicating an intestinal epithelial-intrinsic role for the NAIP/NLRC4 inflammasome in Salmonella limitation (55). Furthermore, immortalized human intestinal epithelial cells (IECs) and primary small intestinal enteroids express minimal levels of NAIP and NLRC4 in comparison to human peripheral blood mononuclear cells (56). This may explain the absence of functional NAIP/NLRC4 inflammasomes in human IECs in these *in vitro* models. The role of NAIP/NLRC4 inflammasomes in the human intestinal epithelium *in vivo* remains unknown. *In vivo*, host or microbial signaling may lead to the upregulation of NAIP/NLRC4 expression in human IECs. Alternatively, NAIP/NLRC4 may be expressed in rare IEC subpopulations not represented in *in vitro* models (57). The question of whether this interesting tissue specificity occurs in the specific group of pregnant women will be the focus of subsequent studies.

## 3 Effects of maternal obesity on inflammation and pyroptosis

Maternal obesity is associated with adverse perinatal outcomes and increased maternal morbidity and mortality. It is a major risk factor for a wide range of antenatal, intrapartum, postpartum, and neonatal complications (58). The release of pro-inflammatory cytokines in the placenta has been linked to maternal obesity and adverse pregnancy complications (59). Both human and animal studies have demonstrated that offspring born to obese mothers are at a higher risk of developing chronic diseases. However, the mechanisms that lead to developmental abnormalities and how different pathways are activated in the offspring of obese mothers remain unclear. Recent evidence suggests that early changes in inflammatory markers may predict the onset of various diseases later in life (60). A study in China was carried out on rats. It found that a high-fat maternal (MHF) diet led to a decrease in the number of peroxisomes in the fetal kidneys. This decrease subsequently activated oxidative stress and inflammasomes, leading to pyroptosis and apoptosis. Downregulation of the peroxisome markers peroxisomes (PEX) 3 and 14 was observed in fetal kidneys, along with a decrease in the antioxidant enzymes Superoxide Dismutase (SOD) 2 and catalase and an increase in the oxidative stress marker Ephx2 (61).Several studies have reported increased levels of free fatty acids (FFA) in the plasma of obese individuals, including palmitic acid (PA), a major saturated fatty acid. PA has been shown to promote inflammatory responses (62). Shirasuna et al. found that PA activates the NLRP3 inflammasome, leading to significant caspase-1 activation and IL-1β secretion. The authors also demonstrated that pyroptosis is involved in this process, as disruption of caspase-1 activity reduced PA-induced IL-1β release (62).

## 4 Pyroptosis and pregnancy-related diseases

### 4.1 Inflammation, toxicity, virus, and miscarriage

Miscarriage represents one of the most prevalent and severe complications of pregnancy. Miscarriage occurs in approximately 15% of recognized pregnancies, and it is associated with substantial costs in terms of physical, psychological, and economic consequences (63). Its occurrence is attributed to a multitude of identified multifactorial etiologies, encompassing fetal chromosomal abnormalities, infections, immunization, thrombosis, endocrine dysfunction, and structural anomalies of the reproductive system (64). Approximately 50% of miscarriages are of unknown etiology (65), and the clinical diagnosis is referred to as recurrent unexplained spontaneous abortion (URSA) (66).

HMGB1 is a non-histone DNA-binding protein that is highly conserved. It is a typical DAMP molecule (67). During pregnancy, high levels of HMGB1 may cause excessive or persistent inflammation, which can lead to unfavorable pregnancy outcomes at critical stages (68). A study conducted in China explored the mechanism by which HMGB1 enters cells through its receptor and activates the necrosis factor kappa B (NF-κB) signaling pathway. This, in turn, activates pyroptosis and NLRP-3 inflammatory vesicles assemble, activating caspase-1 proteins and releasing inflammatory factors, such as HMGB1, inducing aseptic inflammation. Ultimately, this leads to the disruption of the maternal-fetal interface and the development of URSA (68). Furthermore, the activation of NLRP-3 inflammatory vesicles and increased expression of caspase-1 proteins have also been observed in moult and chorionic villus tissues of induced abortion (69).

Environmental toxins can also play a role in causing miscarriage. According to recent studies, exposure to Benzo(a)pyrene (BaP) and its ultimate metabolite benzo(a)pyrene-7,8-dihydrodiol-9,10-epoxide (BPDE) can lead to trophoblast cell pyroptosis and ultimately result in miscarriage. Lnc-HZ14 is highly expressed in human trophoblast cells and RM vs healthy control (HC) chorionic tissues exposed to BPDE, which induces trophoblast cell pyroptosis leading to miscarriage (70).

Maternal viral infections have been a challenging issue for obstetricians for a long time. The Zika virus (ZIKV) is a flavivirus that is transmitted by mosquitoes and infects the female reproductive system (71). Unfortunately, placental infections and vertical transmission of the virus can lead to adverse effects on the fetus, such as congenital Zika virus syndrome (CZS). CZS is a serious condition that can cause microcephaly, intrauterine growth restriction, spontaneous abortions, and developmental abnormalities (72). It has been established that apoptosis may occur in the placenta during ZIKV infection (72). The involvement of pyroptosis, another form of programmed cell death, in this process is still being explored. Researchers have found that ZIKV infection induces pyroptosis of placental trophoblasts through caspase-8-mediated activation of caspase-3 via the exogenous apoptotic pathway. This, in turn, activates GSDME (but not GSDMD), by adding different caspase inhibitors. A study showed that when pregnant mice were infected with ZIKV, those without GSDME had less placental damage and better fetal outcomes (73). In summary, this study has identified a new mechanism of placental damage and congenital CZS caused by ZIKV infection. The virus triggers placental cell pyroptosis through RIG-I recognition of the viral genome, leading to the release of TNF-α. This cytokine activates the cleavage of caspase-8 and caspase-3, which in turn activates the pyroptosis execution factor, GSDME, in placental cells.

In their experiments studying the pathogenesis of silicosis, Kang et al. found that GSDME cutting via Caspases-3/8 was required in addition to Caspase-1/GSDMD. This revealed another important pathway, alongside GSDMD, as a central factor triggering pyroptosis (see **Figure 2**). This mechanism is similar to the one by which ZIKV induces placental damage and CZS (74).

### 4.2 Preterm and full-term labor

Inflammation is a crucial factor in embryo implantation, development, and endometrial metamorphosis (75). Any abnormalities during these stages may result in miscarriage or preterm labor. Preterm labor, which is caused mainly by inflammation leading to premature rupture of membranes (PROM), is a common cause of early birth due to various factors. IL-1 was the first cytokine associated with the mechanism of preterm birth (PTB) linked to infection or acute inflammation in humans, as well as with full-term spontaneous labor (76). In particular, IL-1β induces the expression of cyclooxygenase-2 (COX-2), an enzyme that catalyzes the synthesis of prostaglandins derived from arachidonic acid. These prostaglandins are known to be induced in human pregnancy tissues to promote cervical ripening and myometrial contractions (77), thereby initiating labor. Animal studies have supported the human data, and recent evidence has highlighted the significance of IL-1 in labor for both humans and animals. Inflammatory vesicles are cytoplasmic protein complexes that, once assembled, activate caspase-1 (78). This leads to the release of mature forms of the inflammatory cytokines IL-1β and/or IL-18 (79) and ultimately results in pyroptosis (80). A cross-sectional study conducted in the United States discovered that gasdermin D, the effector molecule of pyroptosis, was detectable in amniotic fluid from patients with spontaneous preterm labor and intra-amniotic infections or aseptic amniotic inflammation (80). However, further experiments are required to investigate the origin of gasdermin D in amniotic fluid. A cross-sectional study found that amniotic fluid and chorionic amnion levels were higher in women who had full-term spontaneous deliveries compared to those who had not delivered. The up-regulation of gasdermin D expression was associated with a corresponding increase in caspase-1 and IL-1β. These findings suggest that pyroptosis is the mechanism responsible for the aseptic inflammatory process in full-term labor (81).

### 4.3 GDM

GDM is a common pregnancy complication (82). It is associated with various adverse pregnancy outcomes, such as an increased risk of gestational infections, amniotic fluid overload, preterm labor, birth injuries, and post-partum infections. Additionally, it can lead to fetal hypoxia, higher-than-normal fetal weights, neonatal hypoglycemia, and macrosomia (83, 84). In GDM, certain alterations result in reduced insulin sensitivity, impaired insulin secretion, and the development of carbohydrate intolerance (85). The molecular mechanisms responsible for these changes remain unclear. Prior research has demonstrated that type 2 diabetes is typified by sustained low-grade inflammation. Furthermore, it has been demonstrated that NLRP3 inflammatory vesicles can contribute to insulin resistance (IR) through the downstream signaling of IL-1β (86). As reported by Ning and colleagues (87), pregnant women with GDM exhibited elevated serum levels of NLRP3, caspase-1, IL-1, and IL-18 in comparison to those with uncomplicated pregnancies. This finding suggests that pyroptosis factors may play roles in the pathogenesis of GDM by promoting chronic inflammation. Hu et al. (88) verified the changes of NLRP3 inflammatory vesicles in GDM placentas. The expression of NLRP3 and Caspase-1 was significantly elevated in GDM placentas relative to healthy placentas. Furthermore, there was a positive correlation between the expression of these proteins and maternal IR, indicating that placental NLRP3 activation is associated with the pathogenesis of GDM. The analysis of potential mechanisms indicates that the activation of NLRP3 inflammatory vesicles may increase the release of IL-1β and IL-18, thereby activating the maternal inflammatory response, IR, and glucose metabolism. Conversely, the irregularities in maternal glucose metabolism may incite the activation of NLRP3 inflammatory vesicles in the placenta, the discharge of IL-1β and IL-18, and exacerbate maternal IR. It can thus be surmised that there is a close relationship between pyroptosis and GDM, which may well represent one of the pathogenic mechanisms of GDM.

It has been demonstrated that there is a direct correlation between GDM and dysfunction of glucose and lipid metabolism involving multiple genes. ROS are harmful mediators of inflammation. There are two primary sources of cellular ROS: phagocytic triphosphopyridine nucleotide (NADPH) oxidase, which produces superoxide anion, and mitochondria, which produce reactive oxidants (62). ROS have a dual role in disease development, acting as both signaling molecules in the cell and unavoidable toxic by-products of aerobic metabolism (89, 90). Recent studies have shown that ROS significantly inhibits NLRP3 inflammasome activation and inflammatory responses both *in vivo* and *in vitro (* 91). TP53-induced glycolysis regulatory phosphatase Gene (TIGAR), a novel p53-inducible protein first identified in 2006 (92, 93), limits ROS and has been demonstrated to contribute to the resolution of ischemia/reperfusion and prevent pathologies such as ischemic diseases (94). The study investigated the impact of TIGAR on placental injury in GDM. The results showed that ATP-induced pyroptosis increased the expression of GSDMD at both the RNA and protein levels. However, cleaved GSDMD proteins were increased after TIGAR knockdown. Based on this evidence, it can be concluded that TIGAR has a mild regulatory effect on the pyroptosis construct compared to ATP (95). Inflammation and oxidative stress in the placenta are crucial factors in GDM. During pyroptosis, oxidative damage and inflammatory cytokines are elevated. The GSDMD pore releases IL-18, IL-1β, and IL-6 upon TIGAR loss, which is often considered the terminal event of pyroptosis (95).

Hyperglycemia induces apoptosis and pyroptosis in vascular endothelial cells, leading to endothelial damage or dysfunction and inflammation (96). The Wnt/β-catenin pathway is considered an important local cell-regulatory signal that determines cell fate and plays a key role in endothelial cell injury (97). β-catenin inhibits macrophage pyroptosis induced by NLRP3 inflammatory vesicles (98). A study conducted in China found that overexpression of spen paralog and direct homolog C-terminal domain containing 1 (SPOCD1) induced by chorionic mesenchymal stem cells (VMSCs) activated the β-catenin pathway, ultimately inhibiting high levels of glucose-induced apoptosis, pyroptosis, and senescence in human umbilical vein endothelial cells (HUVECs) (99).

### 4.4 PE

In healthy pregnancies, regulatory mechanisms at the maternal-fetal interface prevent excessive local and systemic inflammation. However, in cases of PE, these regulatory mechanisms are disrupted due to local hypoxia/ischemia, innate immune activation, hormonal imbalances, and regulatory T-cell abnormalities. This leads to a cytotoxic microenvironment that causes placental inflammation and insufficiency (100). The normal function of trophoblast cells is essential for placental development. Extravillous trophoblasts (EVT) migrate from the trophoblast column and invade the spiral arteries directly through the metaplastic stroma, partially replacing endothelial cells. This process remodels the uterine spiral arteries to increase maternal blood flow to the placenta (101). The pathophysiology of PE is not yet fully understood, but it is believed to involve placental dysplasia, oxidative stress, and altered local and systemic immunomodulation (100). Studies have shown that the trophoblast is a convergence of various pathways (102). The death of trophoblasts and the release of inflammatory factors induced by various stimuli may contribute to the development of PE (102, 103).

**Figure 3:**
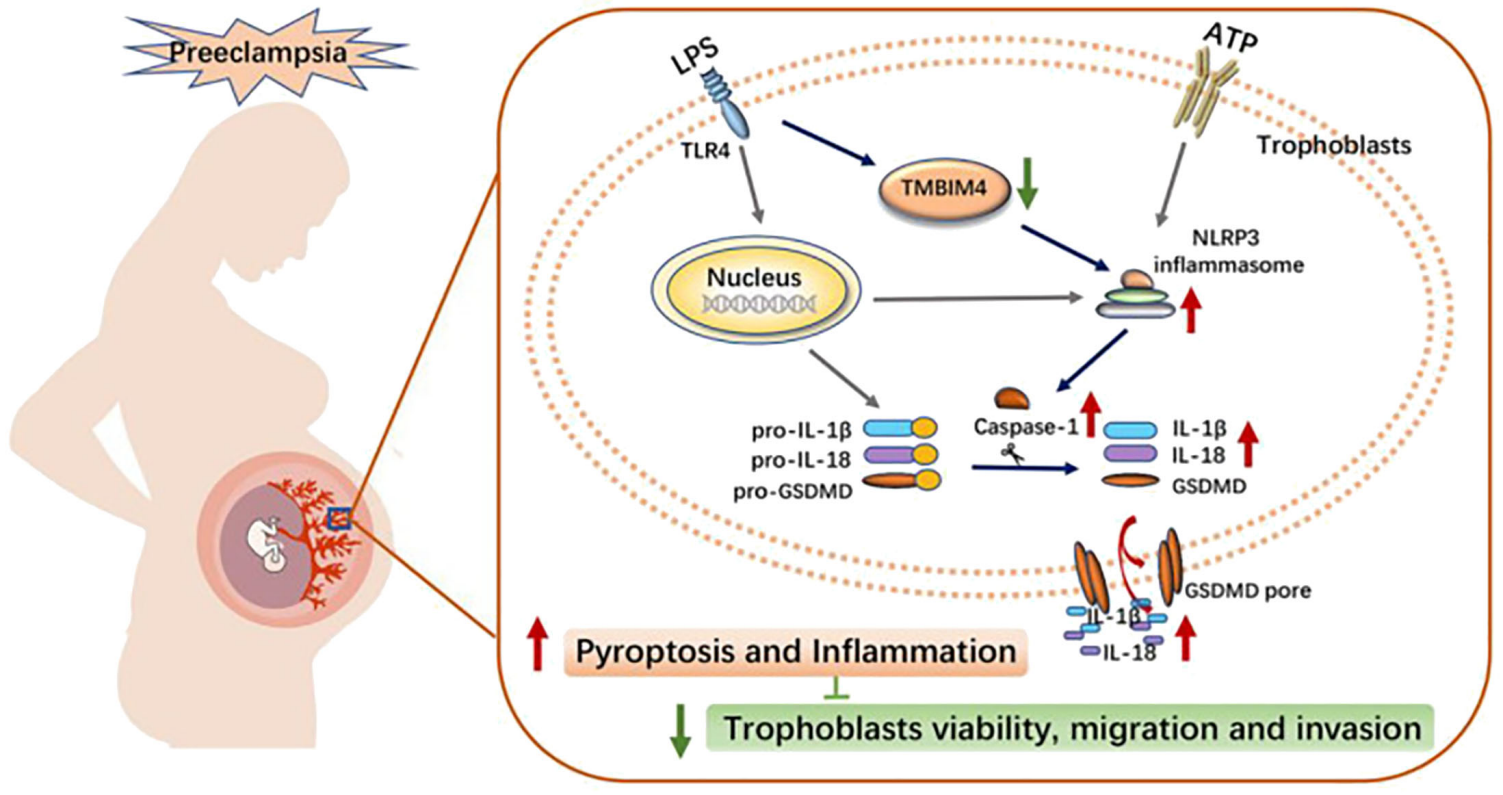
Schematic diagram illustrating the potential mechanism of action of TMBIM4 on PE pathogenesis (104). LPS induced the downregulation of TMBIM4 in the trophoblasts. TMBIM4 deficiency in the trophoblasts markedly enhanced the NLRP3 inflammasome activity and promoted subsequent pyroptosis, thereby disrupting trophoblast viability, migration, and invasion, and might be involved in the pathogenesis of PE. TMBIM4, transmembrane BAX inhibitor motif containing 4. Reproduced with permission from [Liu C], [TMBIM4 Deficiency Facilitates NLRP3 Inflammasome Activation-Induced Pyroptosis of Trophoblasts: A Potential Pathogenesis of Preeclampsia.)]; published by [Biology (Basel], [2023].

A recent study identified a novel protein associated with PE, transmembrane BAX inhibitor motif containing 4 (TMBIM4), which was significantly downregulated in the placentas of women with PE. TMBIM4 is mainly expressed in the human placental trophoblast. Lack of TMBIM4 in the trophoblast cell line significantly enhances NLRP3 inflammatory vesicle activity, promotes subsequent pyroptosis, and ultimately disrupts trophoblast viability, migration, and invasion. This may contribute to the PE pathological process (104). Exposure of trophoblasts to pregnancy-incompatible factors, such as LPS, suppresses TMBIM4 expression. This exacerbates NLRP3 inflammasome-mediated inflammation, leading to trophoblast dysfunction and maternal system syndrome (see **Figure 3**) (104).

MicroRNAs (miRNAs) are small, non-coding, single-stranded RNA molecules that regulate mRNA expression by complementary base pairing with the 3-untranslated region (3-UTR) (105). Certain miRNAs play a crucial role in biological pathways associated with PE, such as the MAPK and TGF-β pathways, and are regulated in PE patients (106). A recent study has observed that miR-124-3p induces apoptosis and reduces cell migration and invasion in trophoblast cells when transfected with HTR8-S/Vneo cells. Additionally, miR-124-3p mimics increased the expression of NLRP3, caspase1 and IL-1β. These results suggest that miR-124-3p plays an important role in preeclampsia by mediating trophoblast cell viability, migration, invasion, and pyroptosis (107).

The clinical manifestations of PE are indicative of systemic inflammation and endothelial dysfunction, resulting in vasoconstriction, end-organ ischemia, and increased vascular permeability (108). According to a study conducted in Pittsburgh, placental cell pyroptosis is the primary sterile inflammatory pathway in e-PE. This may result in the production of virulence factors, such as IL-1β and IL-18, which cause inflammation and enhance the syndrome’s systemic manifestations (100). Therefore, placental cell pyroptosis has been identified as a significant event leading to sterile inflammation in preeclampsia. Upon activation, NLRP3 inflammatory vesicles induce a significant release of mature IL-1β, initiating a positive feedback loop that results in the accumulation of other immune cells such as neutrophils and macrophages, as well as an increase in cytokines and chemokines that pose a risk (45).

Mitochondrial autophagy has been a significant signaling hub for regulating inflammatory cytokine secretion (109). The classical ubiquitin-dependent mitochondrial autophagy pathway in mammals, which is mediated by PTEN-induced putative kinase 1 (PINK1), plays a crucial role in various immune and inflammatory diseases (109). PINK1-mediated mitochondrial autophagy may have a protective role in PE by reducing ROS and trophectodermal pyroptosis (109). A Chinese controlled trial of 20 pregnant women with pre-eclampsia versus 20 healthy pregnant women found that PINK1-mediated mitochondrial autophagy was down-regulated in PE placentas (109), which is consistent with the findings of Chen et al. in a mouse model of PE (110). However, these findings contradict those of Ausman et al. (111), who reported elevated levels of full-length PINK1 relative to inactive PINK1 levels in cleaved PE placentas.

IL11 is a pleiotropic cytokine (112, 113) and is elevated in maternal serum, placenta, and meconium in early preeclamptic pregnancies (114, 115). A large animal study from Australia demonstrated that IL11 drives activation of ASC/NLRP3 inflammatory vesicles, leading to chorioamnionitis, placental and renal fibrosis, and maternal pre-eclampsia syndromes, including chronic postnatal hypertension, in mice. It has also been shown that IL11 has inflammatory vesicle-independent effects leading to dysregulation of trophoblastic differentiation, placental injury, and possibly impaired placental function, resulting in fetal growth restriction and perinatal death (116). It is well established that IL-11 activates many pathways known to be altered in pre-eclampsia, but the exact mechanism by which IL-11 causes placental injury and induces pre-eclampsia is unknown (114), and this finding fills this gap.

### 4.5 Intrauterine growth restriction

Intrauterine growth restriction (IUGR) is defined as the failure of the fetus to reach its genetically determined growth potential *in utero* due to a complex interaction of maternal, placental, fetal, and genetic factors (117).Excessive ROS have been identified as a detrimental factor, with evidence indicating that ROS play a critical role in the onset of cellular death (118, 119). Nuclear factor erythroid-derived 2-related factor 2 (Nrf2) is a transcription factor that plays a pivotal role in the activation of downstream anti-oxidative stress genes (120). and is a crucial mediator of antioxidant pathways (121). An increase in expression has been observed in the cytoplasm of invasive extravillous trophoblast cells, which have been linked to severe early-onset IUGR and preeclampsia (122). Despite evidence indicating a correlation between focal death and IUGR, the underlying molecular mechanisms remain unclear. It was demonstrated that Nrf2 inhibits GSDMD transcription, whereas Nrf2 deficiency upregulates GSDMD expression, thereby exacerbating maternal hypoxia-induced pyroptosis in IUGR offspring. Furthermore, Nrf2 deficiency exacerbates maternal hypoxia-induced Lung dysplasia has been observed in IUGR progeny (123). Additionally, a cross-sectional study by Berna et al. found that GSDMD-mediated cellular pyroptosis was increased in placental tissues in IUGR cases (124). In the rat study, IUGR rats exhibited increased quantification of GSDMD immunofluorescence staining of the hippocampus, increased mRNA and protein expression of NLRP1, caspase-1, and GSDMD, as well as increased quantification of IL-1β and IL-18 in the hippocampus (117). Further clarification is required to determine whether this breakthrough in animal modeling is applicable to humans. The aforementioned studies substantiated the involvement of cellular pyroptosis in the pathogenesis of IUGR, and offered insights into potential avenues for the prevention and management of IUGR.

## 5 Conclusion

**Figure 4:**
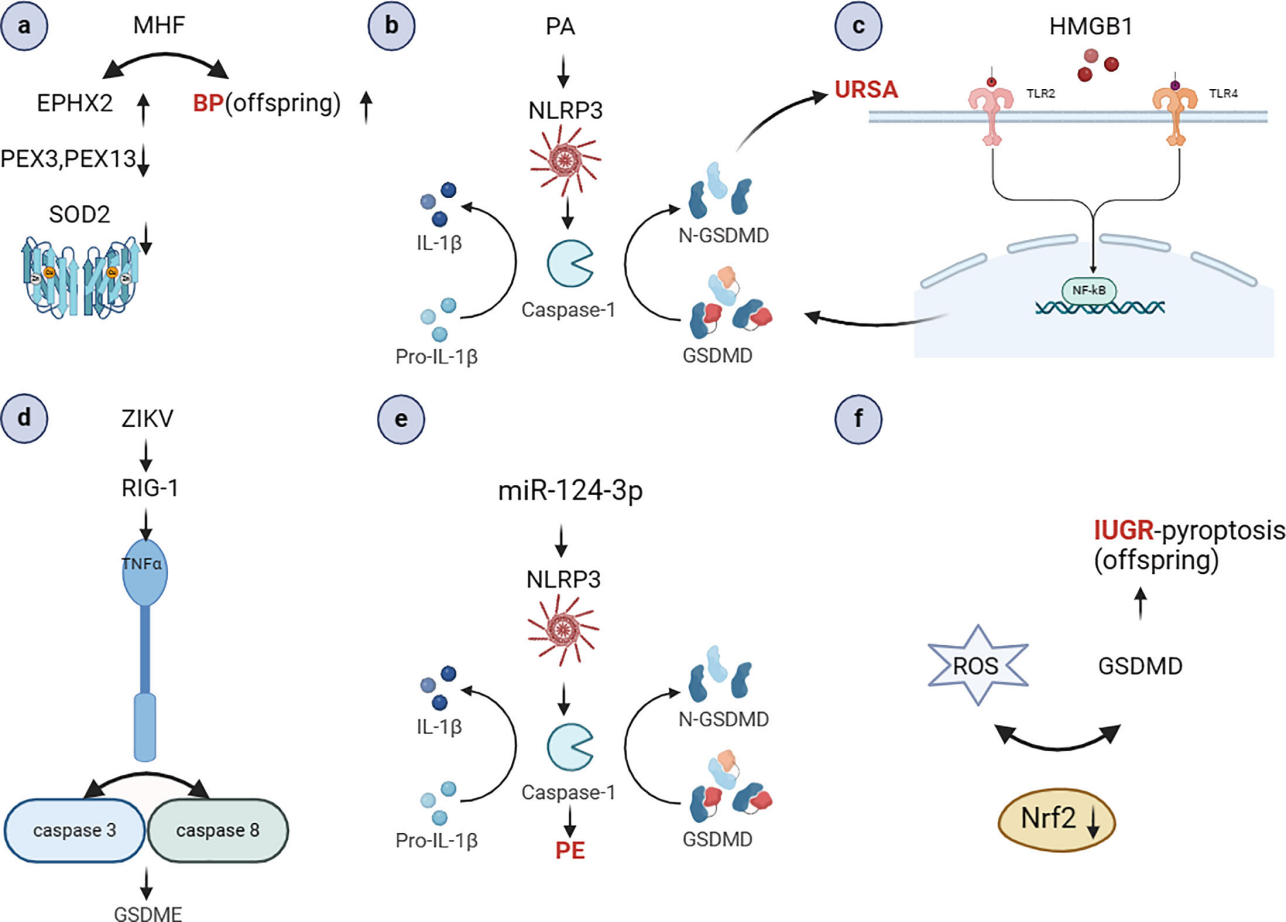
The role of pyroptosis in the occurrence and development of pregnancy-related diseases (Created with BioRender.com). **(A)** MHF may cause high blood pressure in adult offspring. It also lead to the down-regulation of fetal renal peroxisomal markers PEX3 and 14, decreased antioxidant SOD2 and catalase, and elevated oxidative stress marker Ephx2 (61); **(B)** PA can activate NLRP3 inflammatory vesicles, resulting in significant caspase-1 activation and IL-1β secretion (62); **(C)** HMGB1 activates the NF-κB signaling pathway, NLRP-3 inflammatory vesicle assembly, caspase-1 protein activation, and release of inflammatory factors, ultimately inducing aseptic inflammation. This leads to the disruption of the maternal-fetal interface and the development of URSA (68); **(D)** ZIKV infection can activate RIG-I, which recognizes the viral genome and causes placental cell pyroptosis. This leads to the release of TNF-α, which activates caspase-8 and caspase-3, resulting in the cleavage of GSDME in placental cells (73); **(E)** miR-124-3p mimics increased the expression of NLRP3, caspase1 and IL-1β (107); **(F)** Nrf2 deficiency upregulates GSDMD expression, thereby exacerbating maternal hypoxia-induced pyroptosis in IUGR offspring (123). MHF, high-fat maternal; PEX, peroxisomes; SOD2, Superoxide Dismutase 2; HMGB1, high mobility group box-1; ZIKV, Zika virus; Nrf2, Nuclear factor erythroid-derived 2-related factor 2.

Cellular pyroptosis has been extensively documented in the pathogenesis of numerous pathological conditions, including autoimmune disorders, neoplastic growths, and atherosclerosis. Our findings indicate that pregnancy-associated disorders are also associated with pyroptosis to varying aspects (see **Figure 4**). In recent years, the discovery of various pathways has contributed significantly to the development of the field of obstetrics and gynecology. Although the role of pyroptosis in the pathogenesis of abnormal pregnancies has not been fully elucidated, studies have shown that the release of interleukins triggers a series of cascading and causal effects that may lead to an increased prevalence of certain diseases in the offspring, inflammatory effects, labor initiation, and GDM. An integrated analysis of inflammation, immunity and genetics is also essential to gain insight into the role of cellular pyroptosis in the development of pregnancy-related diseases. It is our contention that inflammasome, which play a pivotal role in pyroptosis, represent a promising avenue for the development of novel therapeutic strategies for pregnancy-related disorders. Target exploration has the potential to impede disease progression and enhance human health and well-being.

## Glossary

PE: preeclampsia
GSDMD: Gasdermin D
DAMPs: damage-associated molecular patterns
NET: neutrophil extracellular trap
LPS: lipopolysaccharide
NE: neutrophil elastase
IL-1β: interleukin-1β
PJVK: pejvakin
PAMPs: Pathogen-associated molecular patterns
PRRs: pattern recognition receptors
NLRs: Nucleotide-binding oligomerization domain-like receptors
LRR: leucine-rich repeat
NLRP3: NOD-like receptor pyrin-containing 3
IL-18: interleukin-18
ROS: reactive oxygen species
ASC: apoptosis-associated speck-like protein containing
GSDME: gasdermin E
TF: tissue factor
NINJ-1: ninjurin-1
HMGB1: high mobility group box-1
LDH: lactate dehydrogenase 1
SQSTM1: sequestosome
GDM: gestational diabetes mellitus
RM: recurrent miscarriage
NAIP: apoptosis inhibitory protein
NLRC4: CARD domain-containingprotein 4
IECs: intestinal epithelial cells
MHF: high-fat maternal
PEX: peroxisomes
SOD: Superoxide Dismutase
FFA: free fatty acids
PA: palmitic acid
URSA: unexplained spontaneous abortion
NF-κB: necrosis factor kappa B
BaP: Benzo(a)pyrene
BPDE: benzo(a)pyrene-7,8-dihydrodiol-9,10-epoxide
RM: recurrent miscarriage
HC: healthy control
ZIKV: Zika virus
CZS: Zika virus syndrome
PROM: premature rupture of membranes
PTB: preterm birth
COX-2: cyclooxygenase-2
IR: insulin resistance
NADPH: triphosphopyridine nucleotide
SPOCD1: spen paralog and direct homolog C-terminal domain containing 1
HUVECs: human umbilical vein endothelial cells
EVT: Extravillous trophoblasts
TMBIM4: transmembrane BAX inhibitor motif containing 4
miRNAs: MicroRNAs
3-UTR: 3-untranslated region
PINK1: PTEN-induced putative kinase 1
IUGR: Intrauterine growth restriction
Nrf2: Nuclear factor erythroid-derived 2-related factor 2
